# Perineal Care Strategies and Perineal Outcomes in Primiparous Women: A Prospective Observational Pilot Study

**DOI:** 10.3390/medsci14040462

**Published:** 2026-08-07

**Authors:** Nicole Carchesio, Alessandra D’Angelo, Maria Ilaria Di Giovanni, Cinzia Di Matteo, Annalisa Marotta, Marta Di Nicola, Maria Cristina Curia

**Affiliations:** 1 Complex Operating Structure of Obstetrics and Gynecology Latisana-Palmanova, Azienda Ssanitaria Universitaria Friuli Centrale, Via Sabbionera, 33053 Latisana, Italy; nicole.carchesio@asufc.sanita.fvg.it; 2 Department of Medical, Oral and Biotechnological Sciences “G. D’Annunzio” University of Chieti-Pescara, Via Dei Vestini, 66100 Chieti, Italy; milaria.digiovanni@asl2abruzzo.it (M.I.D.G.); cinzia.dimatteo@asl2abruzzo.it (C.D.M.); annalisa.marotta@unich.it (A.M.); marta.dinicola@unich.it (M.D.N.); mariacristina.curia@unich.it (M.C.C.); 3 Center for Excellence on Ageing and Translational Medicine (CAST), “G. D’ Annunzio” University of Chieti-Pescara, 66100 Chieti, Italy

**Keywords:** antenatal perineal massage, perineal trauma, pelvic floor, vaginal birth, primiparous women, perineal outcomes, postpartum recovery, midwifery care

## Abstract

Background: Pregnancy and childbirth expose the pelvic floor and perineal tissues to substantial biomechanical stress, increasing the risk of obstetric perineal trauma and subsequent maternal morbidity. Objectives: This prospective observational pilot study aimed to explore the association between antenatal perineal massage and perineal outcomes at birth, as well as postpartum pelvic floor findings, in primiparous women. Methods: Women attending an antenatal education programme at the Family Counselling Centre of Francavilla al Mare (Italy) between May and November 2024 were recruited and followed through childbirth and the postpartum period. Results: Twenty-seven primiparous women who experienced spontaneous vaginal birth underwent a comprehensive perineal assessment 6–10 weeks after delivery. Fifteen women (55.6%) self-reported regular antenatal perineal massage during the final weeks of pregnancy, whereas 12 (44.4%) did not perform massage or practiced it only sporadically. Women self-reporting regular perineal massage showed a different distribution of perineal outcomes compared with those in the no/sporadic massage group, with a higher proportion of intact perineum or first-degree tears. Antenatal perineal massage was also associated with a higher frequency of upright birth positions and warm compress use during labour, as well as differences in postpartum vulvar gaping, genital trophism, perineal tone, and pelvic floor muscle performance assessed by the PC-Test. Urinary incontinence and postpartum perineal pain were observed only among women who did not report massage practice. Conclusions: These findings represent exploratory associations and cannot establish causal relationships or demonstrate the independent effect of antenatal perineal massage. Because massage practice clustered with other perineal protection strategies, larger studies with adequate adjustment for potential confounding factors are required to clarify the contribution of individual interventions to perineal and postpartum outcomes.

## 1. Introduction

The pelvic floor is a structure forming the inferior boundary of the abdominal cavity at the bottom and it consists of a bony, muscular, connective and visceral component.

Pregnancy and childbirth have a fundamental influence on anatomy and physiology of the perineal structures. There are three main mechanisms of damage to the pelvic floor related to pregnancy and childbirth: direct mechanical trauma to the muscles, with myogenic damage to the levator ani muscle (LAM) and anal sphincter, neurological damage to the pelvic floor nerves and connective-fascial injury with weakening of the urogenital hiatus [1].

During pregnancy, the pelvic-perineal region is exposed to unfavorable conditions for the normal contraction and relaxation of the Levator ani (LAM). The increasing weight of the gravid uterus exerts direct pressure on the urogenital hiatus, compounded by the anterior shift of the intra-abdominal pressure vector associated with pregnancy-induced lumbar hyperlordosis. As a result, the anterior perineum- an anatomically vulnerable area- is subjected to continuous and abnormal mechanical stress. This results in continuous and abnormal pressure on structurally vulnerable area of the anterior perineum, predisposing it to functional overload and tissue strain [2]. Another particularly critical phase for the pelvic floor occurs during the expulsion of the presenting part. As the fetal head deflects and rotates, it acts as a fulcrum beneath the pubic symphysis, generating marked distension predominantly in the posterior perineum [3]. This mechanism produces an anterior displacement of the perineal body (central fibrous nucleus) and a posterior displacement of the coccyx, leading to significant stretching of the pelvic floor musculature and connective tissues.

Obstetric perineal trauma is common after vaginal childbirth, affecting up to 50% to 90% of women and is associated with significant short- and long-term morbidity, including bleeding, persistent perineal pain, dyspareunia, haematomas, urinary incontinence (up to 47% in the short term) or anal incontinence (up to 17% of gas and/or faecal incontinence), overactive bladder, pelvic organ prolapse, infections and dehiscence, rectovaginal fistulas, vesicovaginal fistulas, pelvic perineal neuropathies, delayed resumption of sexual activity, sexual disorders and depression [4]. The morbidity associated with obstetric perineal trauma can also interfere with the new mother’s ability to care for her newborn and with bonding, with negative repercussions on breastfeeding [5] impacting also on women’s social, sexual, psychological and financial well-being, resulting in social isolation, loss of income and a poorer quality of life [6].

Prevention strategies are a key element in preserving perineal integrity; therefore, the present study focused on perineal massage, the use of warm compresses applied to the perineum, and maternal positioning during the active second stage of labour. Perineal massage is commonly recommended from approximately 35 weeks of gestation onward and has been investigated as a strategy that may influence tissue flexibility, maternal awareness of perineal stretching sensations, and comfort during the second stage of labour. In addition, this practice may enhance maternal body awareness and familiarity with perineal stretching sensations, potentially facilitating controlled relaxation during childbirth [7].

Using warm compresses on the perineum during the active phase of labour showed to improve perineal pain tolerance, reduce maternal stress related to perineal pressure and a decrease of third- and fourth-degree tears was observed, due to increased tissue oxygenation and muscle relaxation [8]. The mother’s freedom of movement and choice of the most comfortable position are recommended. The WHO recommends encouraging women in labour, whether or not they have epidural analgesia, to assume any position they wish, including upright positions [9]. Supine, lithotomy and semi-sitting maternal positions are associated with worse perineal outcomes [10].

This study explored perineal outcomes in women who self-reported regular antenatal perineal massage compared with women who did not perform massage or practiced it only sporadically.

## 2. Study Design

The study was conducted within the Antenatal Course at the Family Counselling Centre in Francavilla al Mare (ASL 2 Lanciano–Vasto–Chieti) between May 2024 and November 2024. During this period, 70 women attending the course received information on perineal health; among them, 51 provided informed consent to participate in the study. The study was conducted in accordance with the principles received approval from the Institutional Ethics Committee of the University “G. d’Annunzio” of Chieti–Pescara (Protocol No. 0001709, date 13 June 2024). 

Of these participants, 43 subsequently gave birth between July 2024 and January 2025 at the Obstetrics and Gynaecology Unit of Chieti. During the postpartum hospital stay, all labour- and delivery-related data were reviewed and collected from the women’s medical records.

Among the 43 women who delivered, 4 were multiparous and 9 underwent caesarean section and were therefore excluded from the analysis. Six to ten weeks after delivery, the remaining 30 women were contacted by telephone to schedule a follow-up evaluation at the Family Counselling Centre in Francavilla al Mare (ASL 2 Lanciano–Vasto–Chieti), where an objective perineal examination was performed. At follow-up, 27 of the 30 women underwent a comprehensive assessment consisting of medical history collection and a detailed objective examination of the perineum, the remaining three women were unavailable because they declined the postpartum visit after initial contact. Data were recorded using a dedicated data collection form. Perineal function was evaluated using the PC-Test, a standardized clinical assessment tool designed to examine postpartum perineal integrity and functional recovery. The PC-Test is a standardized clinical assessment routinely used in pelvic floor rehabilitation in Italy; however, formal psychometric validation studies are currently limited. The instrument assesses voluntary pelvic floor muscle contraction through digital vaginal palpation by evaluating three components: phasic muscle contraction, tonic endurance, and resistance to fatigue. Each component contributes to a total score ranging from 0 to 9, with higher scores indicating better pelvic floor muscle function. For descriptive purposes, scores were categorized as 0–3 (poor function), 4–6 (moderate function), and 7–9 (good function). In addition to the composite score, the examination included assessment of command inversion (inappropriate recruitment of accessory muscles during pelvic floor contraction), perineal tone, vulvar gaping, tissue trophism, pain, and muscle symmetry as separate clinical variables. The PC-Test was performed according to the standardized assessment procedure for those functions.

The final study sample therefore comprised 27 primiparous women aged between 20 and 40 years who experienced spontaneous vaginal delivery. Because the study was conceived as a prospective observational pilot study, the sample was a convenience sample based on eligible women recruited during the predefined study period. No formal a priori sample size calculation was performed. The examiner performing the postpartum assessment was blinded to participants’ self-reported massage status.

Antenatal perineal massage practice was assessed during the postpartum follow-up interview. Women who reported performing massage during the final weeks of pregnancy were included in the massage group, whereas women who did not perform massage or practiced it only sporadically were included in the no/sporadic-massage group. This was a prospective observational pilot study with prospective participant recruitment and follow-up. Exposure (antenatal perineal massage) was assessed at the postpartum visit through maternal self-report, while intrapartum and neonatal variables were extracted from hospital medical records. Regular practice was based on maternal self-report and was not monitored prospectively; detailed information on frequency, duration, adherence, self- versus partner-assisted performance, and standardization of instruction was not systematically collected and is acknowledged as a limitation.

The study included women aged between 18 and 50 years who experienced their first vaginal delivery at the Obstetrics and Gynaecology Unit in Chieti. Exclusion criteria were caesarean delivery, multiparity, and delivery in healthcare facilities other than the Obstetrics and Gynaecology Unit in Chieti.

## 3. Statistical Analysis

Continuous variables were summarized as median and interquartile range (IQR), whereas categorical variables were reported as frequencies and percentages using the group-specific denominator. The primary exploratory endpoint was a favourable perineal outcome at birth, defined as an intact perineum or first-degree tear, compared with an unfavourable outcome, defined as second-degree or higher perineal tear or episiotomy.

Secondary exploratory endpoints included postpartum vulvar gaping, external genital trophism, perineal tone, muscle symmetry, urinary incontinence, postpartum perineal pain, command inversion, and pelvic floor muscle performance assessed using the PC-Test.

Owing to the limited sample size and sparse contingency tables, categorical variables were analysed using exact methods. Two-by-two contingency tables were compared using the two-sided Fisher’s exact test, whereas contingency tables larger than 2 × 2 were analysed using the Fisher–Freeman–Halton exact test. Continuous variables were compared using the Mann–Whitney U test.

For the primary exploratory endpoint, the unadjusted odds ratio (OR) and its 95% confidence interval (95% CI) were calculated to quantify the association between antenatal perineal massage and a favourable perineal outcome.

Given the exploratory nature of this pilot study and the limited sample size, no adjustment for multiple comparisons was performed. Accordingly, *p*-values should be interpreted descriptively and considered hypothesis-generating rather than confirmatory.

Multivariable analyses were not performed because the limited number of participants and outcome events did not allow reliable adjustment for clinically relevant confounders without substantial risk of model overfitting.

All statistical tests were two-sided, and given the exploratory nature of this pilot study and the absence of adjustment for multiple comparisons, *p*-values are reported descriptively and should be interpreted as measures of compatibility with the observed data rather than as confirmatory evidence.

Statistical analyses were performed using R 4.5.2 (R Foundation for Statistical Computing, Vienna, Austria).

## 4. Results

The sample consisted of 27 women at term, with cephalic presentation, aged between 20 and 40 years, with a median age of 33 years. The most frequently adopted maternal position during childbirth was the lithotomy position, used by 12 women (44.4%), followed by the all-fours position in 6 women (22.2%) and the crouching position in 5 women (18.5%). The remaining 4 women (14.8%) delivered in lateral or standing positions (Table 1).

An association was observed between antenatal perineal massage practice and the distribution of birth positions. Women who performed perineal massage were more likely to give birth in free positions, particularly crouching and all-fours positions.

No statistically significant differences were observed between the perineal massage and no-massage groups regarding epidural analgesia use (exact *p* = 0.236), induction methods (exact *p* = 0.428), catheterization (exact *p* = 0.452), or gestational age at delivery (exact *p* = 0.964). Significant differences were found regarding warm compresses, which were more frequently used among women who performed perineal massage (exact *p* = 0.007) (Table 1). The study also investigated two indicators of neonatal well-being at birth: the Apgar score and umbilical cord blood pH. Arterial cord blood pH samples were routinely performed on all newborns who participate in the study employing a standardised sampling protocol. Most newborns achieved an Apgar score of 9/10 at birth (85.2%), while two newborns (7.4%) had a score of 8/9. One newborn (3.7%) scored 8/8 and another (3.7%) scored 7/8. The median umbilical artery pH value was 7.26, with an interquartile range of 7.22–7.29 (Table 1). The vulvar gaping (Table 2), classified as G1 (22–23 mm) and G2 (23–33 mm), reflects the tone of the superficial perineum, with lower laxity indicating greater tone. Overall, 70.4% (n = 19) of postpartum women presented with G1, while 29.6% (n = 8) had G2. A reduced vulvar gaping (G1), indicative of better superficial perineal support, was observed more frequently in the massage group than in the no-massage group (exact *p* = 0.008). As shown in Table 2, external genital trophism, indicative of tissue oxygenation and perineal health, was good in 51.9% (n = 14) of women, moderate in 29.6% (n = 8), and poor in 18.5% (n = 5).

**Figure 1:**
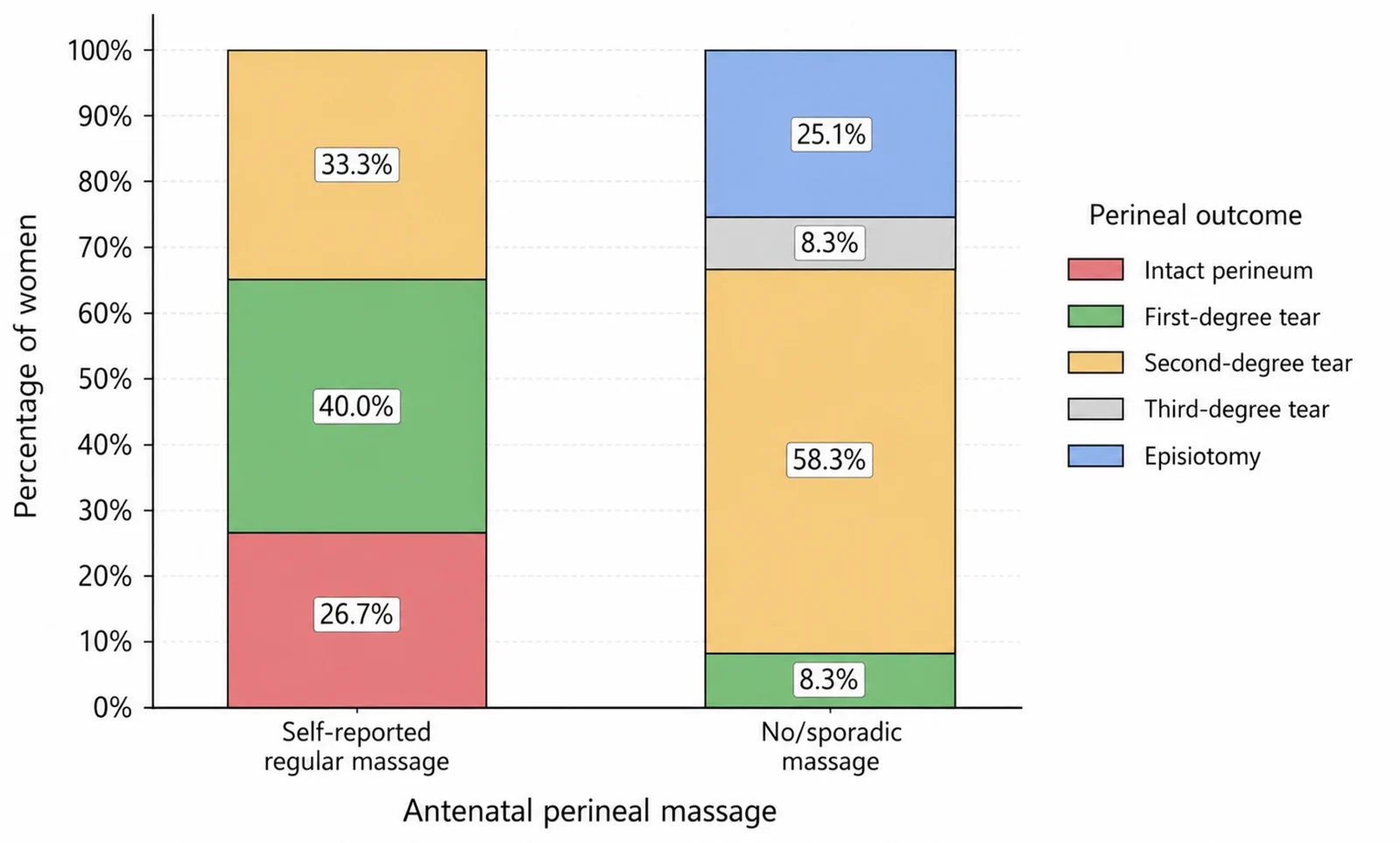
Distribution of perineal outcomes in relation to antenatal perineal massage practices.

Good external genital trophism was predominantly observed among women who practiced perineal massage (*p* < 0.001), whereas moderate or poor trophism was considerably more common among those who did not. Perineal tone assessed via the cough reflex showed good tone in 44.4% (n = 12), moderate tone in 25.9% (n = 7), and poor tone in 29.6% (n = 8) of women. It also differed significantly between groups (*p* < 0.001). All women presenting good perineal tone at postpartum evaluation had performed antenatal perineal massage, while poor tone was mainly observed among women who had not practiced massage (88%). Pelvic floor muscle symmetry on palpation was observed in 96.3% (n = 26) of the sample and did not differ significantly between groups (exact *p* = 0.444) (Table 2). Stress urinary incontinence was reported by 22.2% (n = 6) of puerperas and was reported only in the no-massage group (exact *p* = 0.003). As shown in Table 2, perineal pain was absent in 81.5% (n = 22) of women, whereas 18.5% (n = 5) presented trigger points, predominantly in association with vaginal–perineal sutures. Urinary incontinence and perineal pain were observed exclusively among women who did not perform antenatal perineal massage. Stress urinary incontinence affected 22.2% of the overall sample and was reported only in the no-massage group (*p* = 0.003). Similarly, all cases of postpartum perineal pain occurred among women who had not practiced massage (exact *p* = 0.010). Pelvic floor muscle function assessed by the PC-Test differed between groups (Table 2). High PC-Test scores (7–9) were more frequently observed among women reporting regular antenatal perineal massage, whereas low scores (0–3) were more common among women in the no/sporadic massage group (exact *p* = 0.002). Command inversion was also less frequently observed in the massage group (exact *p* = 0.002). The test was performed by instructing the woman to contract the pubococcygeal muscle while two flat fingers were placed 2–3 cm distal to the posterior vaginal wall. Scores ranged from 0 to 9 and were classified into three categories: 0–3, 4–6, and 7–9. A score of 7–9 was obtained by 51.9% (n = 14) of women, followed by 25.9% (n = 7) scoring 0–3 and 22.2% (n = 6) scoring 4–6. Women reporting self-reported regular antenatal perineal massage more frequently achieved higher PC-Test categories. (exact *p* = 0.002). High PC-Test scores (7–9), were achieved predominantly by women in the massage group (86%), whereas low scores (0–3) were mainly observed among women who did not perform massage (86%) (Table 2). The PC-Test also evaluates command inversion, defined as the involuntary recruitment of accessory muscle groups during pelvic floor contraction. Command inversion was less frequent among women who practiced massage (exact *p* = 0.002), indicating a lower frequency of accessory muscle recruitment during pelvic floor contraction among women in the massage group. In 70.4% (n = 19) of women, no command inversion was observed, indicating isolated pelvic floor activation. Among the remaining participants, 18.5% (n = 5) showed antagonistic contraction of the gluteal muscles alone, while 11.1% (n = 3) demonstrated simultaneous contraction of the gluteal and abdominal muscles, likely reflecting limited pelvic floor proprioception. Perineal massage in the last weeks of pregnancy was performed as a constant and habitual practice by 15 women (55.6%). The remaining 12 women (44.4%) did not perform it or did so in a sporadic manner. Among women who practiced perineal massage, 27% maintained an intact perineum, 40% experienced only a first-degree tear and 33% experienced a second-degree tear, whereas no cases of episiotomy or third-degree tears were observed (Figure 1).

The figure shows perineal outcomes in the two study groups. (Self-reported regular massage) 15 women performed antenatal perineal massage during the last weeks of pregnancy as a habitual practice, whereas the remaining (No/sporadic massage) 12 women, either did not perform perineal massage or did so only sporadically. Perineal outcomes are presented as percentages within each group and include intact perineum, first-degree tear, second-degree tear, third-degree tear, and episiotomy.

In contrast, among women who did not perform perineal massage, second-degree tears represented the most frequent outcome (58%), while episiotomies represented 25% of cases and the only third-degree tear in the cohort occurred exclusively in this group, first-degree tear was only observed in 8% of cases (Figure 1).

Considering the predefined primary exploratory endpoint (intact perineum or first-degree tear versus second-degree tear, episiotomy or third-degree tear), women who self-reported antenatal perineal massage had higher unadjusted odds of a favourable perineal outcome than women who did not perform massage or performed it only sporadically (unadjusted OR 22.0, 95% CI 2.18–221.98; exact *p* = 0.005).

To further explore whether these postpartum functional variations translated into differences in childbirth outcomes, perineal outcomes were examined according to antenatal perineal massage practices. Neither Apgar score nor umbilical cord blood pH was significantly associated with antenatal perineal massage practice (*p* = 0.581 and *p* = 0.850, respectively), suggesting that neonatal condition at birth was comparable between groups within this study population. Although both measures are widely used to assess neonatal condition at birth, they provide complementary rather than interchangeable information. The Apgar score reflects the newborn’s clinical adaptation to extrauterine life by assessing heart rate, respiratory effort, muscle tone, reflex irritability, and colour, whereas umbilical cord blood pH provides an objective biochemical assessment of the fetal acid–base status at delivery and is considered a marker of intrapartum hypoxia. Previous research has demonstrated only a weak correlation between Apgar scores and umbilical arterial cord blood acid–base parameters, indicating that these measures capture different aspects of neonatal condition and should therefore be interpreted together rather than as substitutes. In particular, Młodawska et al. [11] reported a low but observed association between Apgar scores and umbilical cord blood pH, concluding that the clinical assessment of neonatal adaptation and biochemical evaluation of acid–base balance provides complementary information for the assessment of newborn well-being. The median neonatal was 3350 g, with an IQR of 3000–3580 g. An observed association was found between vulvar gaping and perineal outcome (*p* < 0.020), with women experiencing more favourable outcomes (intact perineum or first-degree tear) more frequently presenting a reduced gaping (G1). External genital trophism was good in all women with an intact perineum, in 85.7% (n = 6) of women with first-degree tears, and in 33.3% (n = 4) of those with second-degree tears. Conversely, poor trophism was observed in 33.3% of women with second-degree tears, 66.7% of women who underwent episiotomy, and in the woman with a third-degree tear. Asymmetry was observed only in the woman who experienced a third-degree tear, who did not perform perineal massage. High PC-Test scores (7–9) were recorded in all women with an intact perineum, in 71.4% (n = 5) of women with first-degree tears, and in 33.3% of women with second-degree tears and episiotomy. Low scores (0–3) were most frequent among women with second-degree tears (33.3%), episiotomy (66.7%), and the third-degree tear. All women with an intact perineum and 85.7% (n = 6) of those with first-degree tears showed no command inversion.

## 5. Discussion

Obstetric perineal trauma is associated with substantial short- and long-term morbidity and may adversely affect a woman’s ability to care for her newborn, interfere with early mother–infant bonding and negatively impact breastfeeding. Severe perineal trauma, particularly when occurring in the context of dystocic or operative vaginal delivery, as well as the experience of perineal suturing itself, may be perceived as traumatic childbirth events. These experiences are often recalled with discomfort or psychological distress [5,12].

In this prospective observational pilot study, women who self-reported regular antenatal perineal massage showed a more favourable distribution of perineal outcomes at birth and better postpartum perineal and pelvic floor findings compared with women who did not perform massage or practiced it only sporadically. These findings should be interpreted as exploratory associations rather than evidence of effectiveness.

The distribution of perineal outcomes suggested a shift towards less severe trauma among women who practiced massage, although the small sample size and sparse outcome counts require cautious interpretation.

Previous studies have suggested that warm compresses may enhance tissue elasticity, improve local blood flow, reduce perineal pain, and decrease the risk of severe obstetric perineal trauma by promoting gradual tissue stretching during fetal descent [13]. In our study, warm compresses were used in 63% of women overall and a higher frequency was observed among women who had performed antenatal perineal massage.

Notably, all women who maintained an intact perineum had benefited from the use of warm compresses. This finding may indicate that women who actively engaged in antenatal perineal preparation were also more likely to benefit from evidence-based intrapartum perineal protection measures. Although the independent effect of warm compresses on perineal outcomes could not be determined due to the limited sample size, women exposed to both interventions appeared to experience a more favourable distribution of perineal outcome. Although antenatal perineal massage was associated with other intrapartum protective practices, including upright birth positions and warm compresses, the independent contribution of each intervention could not be disentangled in this observational sample. Labour was induced and/or augmented with synthetic oxytocin in 37% of the sample.

No statistically significant association was observed between synthetic oxytocin use and perineal massage. However, previous literature suggests that, particularly in cases of prolonged labour, the administration of synthetic oxytocin is associated with an increased risk of anal sphincter injury. This association may be explained by the fact that synthetic oxytocin infusion, together with maternal sympathetic activation, may contribute to sustained pelvic floor muscle contraction.

Epidural analgesia was administered in 70.4% of women and was not significantly associated with perineal massage. This finding is consistent with the results reported by Loewenberg-Weisband et al. [14], who attributed the increased risk of perineal tears to a prolonged second stage of labour rather than to epidural anaesthesia per se. Similarly, Simic et al. [15] demonstrated that prolongation of the second stage of labour is associated with an increased risk of severe perineal injury. This pattern was also observed in our sample, in which women experiencing prolonged labour showed a higher incidence of perineal tears. Although epidural analgesia effectively reduces pain perception, it may impair a woman’s ability to perceive the urge to push, potentially prolonging the expulsive phase and indirectly influencing perineal outcomes.

Furthermore, higher PC-Test scores were observed among women with more favourable perineal outcomes and were also associated with reported antenatal perineal massage practice.

Specifically, 52% of puerperae achieved scores between 7 and 9, indicating good muscle function, achieved by 86% of women who had performed massage. In contrast, scores between 0 and 3 were predominantly observed among women with less favourable perineal outcomes (67% of those who underwent episiotomy and the woman who sustained a third-degree tear), predominantly observed among women who had not practiced massage (86%).

Urinary incontinence was reported in 22.2% of puerperae overall, specifically in 33.3% of women with a second-degree tear, 33.3% of those who underwent episiotomy, and in the woman who sustained a third-degree tear. Particularly noteworthy is the observation that all cases of urinary incontinence occurred among women who had not performed antenatal perineal massage. Likewise, all cases of postpartum perineal pain were recorded in the sample who did not perform perineal massage. Although these findings should be interpreted cautiously due to the observational design and small sample size, they suggest a potential association between perineal integrity at birth and improved postpartum pelvic floor health. These findings are consistent with the data reported by Mosca et al. [6], which underscore the impact of perineal trauma on long-term pelvic floor function.

Moreover, the association between an intact perineum and higher PC-Test scores is consistent with a potential role of preventive perineal care strategies, such as antenatal perineal massage and the adoption of upright or free birthing positions during labour; however, the present design does not allow attribution of an independent protective effect to any single strategy.

This study has several important limitations. First, the final sample included only 27 women, which limited statistical power and produced sparse cells for several clinically relevant outcomes. The study was therefore underpowered to assess rare but important endpoints such as OASIS, episiotomy, urinary incontinence, and postpartum pain. Second, this was a single-centre observational pilot study based on a convenience sample, and no causal conclusions can be drawn. Third, antenatal perineal massage was associated with other intrapartum practices, particularly upright/free birth positions and warm compresses [16,17], suggesting relevant confounding by a broader pattern of evidence-based childbirth care and maternal engagement. Fourth, the small number of participants and events did not allow reliable multivariable adjustment. Finally, detailed information on massage frequency, duration, adherence, standardized instruction, self- versus partner-assisted practice, and some intrapartum management variables was not systematically collected. Therefore, the findings should be considered exploratory and hypothesis-generating and require confirmation in larger multicentre studies with longer follow-up.

## 6. Conclusions

In this prospective observational pilot study, unadjusted associations were observed between self-reported aqantenatal perineal massage, perineal outcomes at birth, and postpartum pelvic floor findings. The perineum plays an important role during childbirth, and strategies aimed at supporting perineal health represent an important component of woman-centred obstetric care. However, these findings should be interpreted with caution. The small sample size, observational design, and clustering of perineal massage with other intrapartum practices, including warm compresses and upright/free birth positions, do not allow causal conclusions or attribution of observed outcomes to massage alone. Further larger multicentre studies with standardized assessment of exposure, predefined outcomes, and appropriate adjustment for potential confounding factors are required to clarify the independent contribution of antenatal perineal massage and other perineal care strategies. These findings may contribute to generating hypotheses for future research and to improving understanding of perineal care approaches within midwifery practice.

## Figures and Tables

**Table 1:** Intrapartum characteristics according to antenatal perineal massage practice.

Variable	Category	Overall N = 27	Perineal Massage n = 15	No/Sporadic Massage n = 12	Exact Two-Sided *p*-Value
Epidural analgesia	Yes	19/27 (70.4%)	9/15 (60.0%)	10/12 (83.3%)	0.236
	No	8/27 (29.6%)	6/15 (40.0%)	2/12 (16.7%)	
Birth position	Crouching	5/27 (18.5%)	5/15 (33.3%)	0/12 (0.0%)	0.016
	All-fours	6/27 (22.2%)	5/15 (33.3%)	1/12 (8.3%)	
	Standing	2/27 (7.4%)	1/15 (6.7%)	1/12 (8.3%)	
	Lateral	2/27 (7.4%)	0/15 (0.0%)	2/12 (16.7%)	
	Lithotomy	12/27 (44.4%)	4/15 (26.7%)	8/12 (66.7%)	
Warm compresses	Yes	17/27 (63.0%)	13/15 (86.7%)	4/12 (33.3%)	0.007
	No	10/27 (37.0%)	2/15 (13.3%)	8/12 (66.7%)	
Induction	Prostaglandins	11/27 (40.7%)	6/15 (40.0%)	5/12 (41.7%)	0.428
	Oxytocin	10/27 (37.0%)	4/15 (26.7%)	6/12 (50.0%)	
	CRB	1/27 (3.7%)	1/15 (6.7%)	0/12 (0.0%)	
	No	5/27 (18.5%)	4/15 (26.7%)	1/12 (8.3%)	
Catheterization	Yes	11/27 (40.7%)	5/15 (33.3%)	6/12 (50.0%)	0.452
	No	16/27 (59.3%)	10/15 (66.7%)	6/12 (50.0%)	
Gestational age	37/37 + 6	1/27 (3.7%)	1/15 (6.7%)	0/12 (0.0%)	0.964
	38/38 + 6	1/27 (3.7%)	1/15 (6.7%)	0/12 (0.0%)	
	39/39 + 6	8/27 (29.6%)	5/15 (33.3%)	3/12 (25.0%)	
	40/40 + 6	9/27 (33.3%)	4/15 (26.7%)	5/12 (41.7%)	
	41/41 + 6	8/27 (29.6%)	4/15 (26.7%)	4/12 (33.3%)	
Apgar score	7/8	1/27 (3.7%)	0/15 (0.0%)	1/12 (8.3%)	0.581
	8/8	1/27 (3.7%)	0/15 (0.0%)	1/12 (8.3%)	
	8/9	2/27 (7.4%)	1/15 (6.7%)	1/12 (8.3%)	
	9/10	23/27 (85.2%)	14/15 (93.3%)	9/12 (75.0%)	

**Table 2:** Postpartum perineal characteristics and pelvic floor assessment according to antenatal perineal massage practice.

Variable	Category	Overall N = 27	Perineal Massage n = 15	No/Sporadic Massage n = 12	Exact Two-Sided *p*-Value
Vulvar gaping	G1	19/27 (70.4%)	14/15 (93.3%)	5/12 (41.7%)	0.008
	G2	8/27 (29.6%)	1/15 (6.7%)	7/12 (58.3%)	
Trophism	Good	14/27 (51.9%)	13/15 (86.7%)	1/12 (8.3%)	<0.001
	Moderate	8/27 (29.6%)	1/15 (6.7%)	12 (58.3%)	
	Poor	5/27 (18.5%)	1/15 (6.7%)	4/12 (33.3%)	
Perineal tone	Good	12/27 (44.4%)	12/15 (80.0%)	0/12 (0.0%)	<0.001
	Moderate	7/27 (25.9%)	2/15 (13.3%)	5/12 (41.7%)	
	Poor	8/27 (29.6%)	1/15 (6.7%)	7/12 (58.3%)	
Muscle symmetry	Yes	26/27 (96.3%)	15/15 (100.0%)	11/12 (91.7%)	0.444
	No	1/27 (3.7%)	0/15 (0.0%)	1/12 (8.3%)	
Urinary incontinence	Yes	6/27 (22.2%)	0/15 (0.0%)	6/12 (50.0%)	0.003
	No	21/27 (77.8%)	15/15 (100.0%)	6/12 (50.0%)	
Pain	Mild	5/27 (18.5%)	0/15 (0.0%)	5/12 (41.7%)	0.010
	Absent	22/27 (81.5%)	15/15 (100.0%)	7/12 (58.3%)	
Command inversion	Glutes	5/27 (18.5%)	0/15 (0.0%)	5/12 (41.7%)	0.002
	Glutes and abdomen	3/27 (11.1%)	1/15 (6.7%)	2/12 (16.7%)	
	No	19/27 (70.4%)	14/15 (93.3%)	5/12 (41.7%)	
PC-Test	0/3	7/27 (25.9%)	1/15 (6.7%)	6/12 (50.0%)	0.002
	4/6	6/27 (22.2%)	2/15 (13.3%)	4/12 (33.3%)	
	7/9	14/27 (51.9%)	12/15 (80.0%)	2/12 (16.7%)	

## Data Availability

The original contributions presented in this study are included in the article. Further inquiries can be directed to the corresponding author.
